# A Case of Reversible Cerebral Vasoconstriction Syndrome in a Healthy Adult Male

**DOI:** 10.7759/cureus.8374

**Published:** 2020-05-31

**Authors:** Alysha Roberts, Nicholas Sowers

**Affiliations:** 1 Emergency Medicine, Dalhousie University, Halifax, CAN; 2 Emergency Medicine, Queen Elizabeth II Health Science Center, Halifax, CAN

**Keywords:** rcvs, reversible cerebral vasoconstriction syndrome, emergency department

## Abstract

Reversible cerebral vasoconstriction syndrome (RCVS) represents a potentially under-recognized cause of thunderclap headache in patients presenting to the ED. While a rarely made diagnosis in emergency medicine practice, RCVS may be as common as subarachnoid hemorrhage (SAH). RCVS typically presents as a sudden onset, excruciating headache that may be associated with nausea, vomiting, photophobia, or other features with overlap in the clinical presentation of both SAH and migraine headaches. As a result of historical features overlapping the presentation of SAH, particularly the rapidity of onset and peak of severity, these patients are typically investigated for SAH and when that workup ultimately is reassuring, clinicians may often misattribute RCVS symptoms as migrainous.

We present a case of a 35-year-old healthy male who presented with a severe, sudden onset headache, nausea, and photophobia to the ED on four occasions within a nine-day period. He was initially investigated appropriately for SAH; receiving an unenhanced head CT and lumbar puncture, which were both unremarkable. Following this initial workup, he was assessed on several other occasions, treated symptomatically as a migraine, and discharged home. On the fourth ED visit a CT angiogram (CTA) was completed that demonstrated the characteristic “string of beads” appearance of the middle cerebral artery (MCA) diagnostic of RCVS. This case describes the key features and investigations of a patient with RCVS and highlights the importance of early and accurate diagnosis of thunder clap headache in which SAH has been excluded.

## Introduction

In the ED, patients with severe headaches are evaluated for red flags, risk factors, and any symptoms that may support the diagnosis of a serious cause of headache [i.e., subarachnoid hemorrhage (SAH), meningitis]. For patients in which SAH has been ruled out, reversible cerebral vasoconstriction syndrome (RCVS) represents an underdiagnosed presentation of severe, sudden onset (thunder clap) headache [1]. Though a majority of RCVS cases result in a benign clinical course, a proportion of patients may go on to develop serious complications such as intracranial hemorrhage, seizures, or cerebral infarcts secondary to RCVS [1-3].

Reversible cerebral vasoconstriction syndrome is more common in women, typically in their 40s-50s who have a history of migraine [4-5]. In the majority of cases, there exists an identifiable trigger such as exertion, coughing, defecation, or the use of vasoactive substances [3-4]. Several scoring systems have been developed to help with timely diagnosis [5-6], as patients endure an estimate of 9.3 days of symptoms and may seek care up to six times before receiving a diagnosis [7-8]. Once a diagnosis is made, the treatment of RCVS is largely supportive [9-10]. While the use of oral calcium channel blockers such as nimodipine and verapamil has been studied, there remains limited evidence of their effectiveness [11-12]. 

In this case, we present a healthy young male who was diagnosed with RCVS in the ED after four visits and initially receiving a diagnosis of migraine. This case illustrates the common clinical features of RCVS, including nausea, vomiting, photophobia, and recurrent severe headaches, as well as the pertinent investigations required to make the diagnosis in an otherwise healthy and uncommon population.

## Case presentation

Patient information

The patient was a 35-year-old Caucasian male who presented to the ED with a chief complaint of a sudden onset, severe headache recurring over several days and leading to significant functional impairment. His medical history was significant for painless ocular migraines and otherwise unremarkable. He described a paternal history of a SAH leading to a generalized seizure several years ago. The patient smokes 8-10 cigarettes per day in addition to using approximately 0.5 g of cannabis daily.

In the week leading up to the visit described in this case, the patient had presented to the ED three times with a chief complaint of a severe and sudden onset headache. Over the nine-day period of headaches, the pain became progressively worse. The headaches were associated with neck pain, vomiting, and photophobia without other visual abnormalities. The patient denied fever, chills, and any neurological deficits. Serial neurological examinations in the ED were unremarkable.

Initially, the patient presented to a peripheral ED. On this occasion, he received metoclopramide, ketorolac, and acetaminophen with codeine for pain management. An unenhanced head CT was performed approximately 24 hours after symptom onset, which revealed no significant findings.

On his second presentation with a recurring headache five days later, the patient received similar treatment for pain and was discharged with a diagnosis of migraine. A lumbar puncture performed on his third presentation the following day (seven days since symptom onset) was unremarkable. Finally, at his fourth presentation (nine days since initial onset), his progressive headache remained typical for a SAH and was not in keeping with a post-lumbar puncture headache. As such, a CT angiogram (CTA) was conducted, which confirmed a diagnosis of RCVS. See Table 1 for a detailed timeline.

**Table 1:** Timeline of presentation and investigations leading to RCVS diagnosis. RCVS, reversible cerebral vasoconstriction syndrome

Date	Investigations/Treatment
07/01/2019	Unenhanced CT, pain management
12/01/2019	Pain management
13/01/2019	Lumbar puncture, pain management
15/01/2019	Second unenhanced CT, CT angiogram, pain management

Clinical findings

On exam, the patient was in no apparent distress with stable vital signs. His Glasgow Coma Scale (GCS) score was 15/15. His neurological exam was unremarkable, with no apparent cranial nerve abnormalities. His pupils were equal and reactive to light. Power and sensation were normal and equal bilaterally in the upper and lower extremities. He had no gait abnormalities or cerebellar signs. He had no signs of meningeal irritation.

Diagnostic assessment

The patient received an unenhanced head CT on his initial presentation to rule out SAH. The CT scan showed no intracranial hemorrhage, edema, mass, or signs of herniation. No acute infarct was evident. A lumbar puncture was performed on the third visit to rule out meningitis. Cerebrospinal fluid (CSF) analysis indicated normal appearing CSF with protein and glucose levels within normal range. Table 1 illustrates the sequence of presentation and investigations the patient received. 

**Figure 1:**
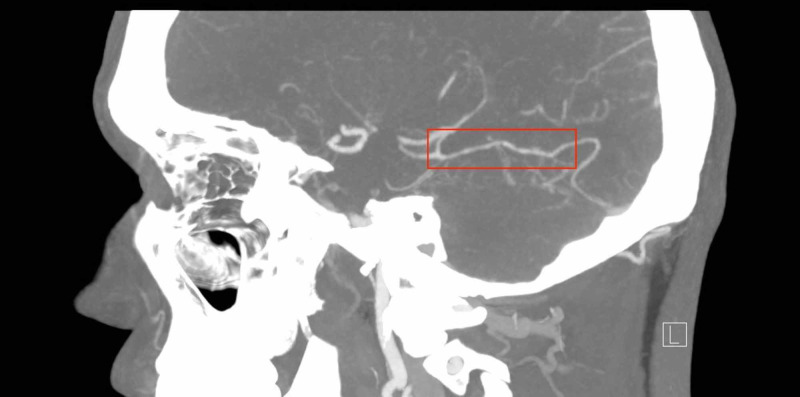
Sagittal view of the "string of beads" appearance indicative of vasospasm, as seen on CTA. CTA, CT angiogram

A second CT scan performed on his fourth visit revealed no changes from his initial presentation. At this point, a CTA was ordered to investigate potential vascular causes of his recurrent headaches, including arterial dissection. The results of the angiogram confirmed the absence of dissection, stenosis, intracranial aneurysm, or occlusion. Multiple focal narrowings were present in the posterior branches of the right middle cerebral artery territory, which were consistent with a RCVS. No other areas of focal narrowing were present. Figure 1 highlights the area of vasoconstriction seen on CTA that was diagnostic in this case. 

Neurology was consulted to assist in patient management. He was subsequently treated with supportive management and made an uneventful recovery.

## Discussion

This case illustrates the key features of RCVS in an otherwise healthy young male and the challenges of making a timely diagnosis. In this case, the time from first symptoms to diagnosis was consistent with previous literature suggesting an average time of nine days to receive a diagnosis [7-8]. As RCVS presents similarly and with equal frequency to SAH [9], it is important to consider it during the early stages of workup for SAH in the ED. 

In this case, features including nausea, vomiting, and photophobia were present. Though these are also often seen in migraines, RCVS is distinct in that the presenting headache is excruciating and abrupt in nature [6]. Other features that should prompt the consideration of RCVS include one or more recurrent thunderclap headaches, or a sudden increase in headache intensity [4]. Early differentiation from migraine is particularly important. Common migraine treatments such as triptans may worsen symptoms, and in some cases have been documented as inducing RCVS [13]. 

Additionally, the patient had a history of background migraines and use of vasoactive substances (i.e., daily cannabis use), both of which have been documented as factors associated with RCVS [3, 6-7]. This patient did not fit with the typical demographic of RCVS, which is most commonly reported in women in their 40s-50s [3, 5, 8]. This case demonstrates an earlier age of presentation than is typically seen in RCVS. Further documentation of cases in younger males is important to determine whether the features of RCVS differ by gender. 

Although this patient had some historical features that are documented to be associated with RCVS (e.g., vasoactive substance use, migraine), it is not clear whether they were temporally related to the headache. Another important feature that has not been documented in the literature is the patient's family history of a parental thunderclap headache and suspected SAH. While the full details surrounding this incident could not be recounted by the patient, it is possible that this positive family history was also contributory to our patient's presentation. Future studies should explore whether patients with a family history of thunderclap headache are at higher risk of RCVS. 

## Conclusions

Reversible cerebral vasoconstriction syndrome remains an underdiagnosed presentation of thunderclap headache to the ED, but is an important consideration in cases where SAH has been ruled out. The present case highlights the importance of considering RCVS in all demographics in cases where other risk factors such as vasoactive substance use or a significant family history have been documented.
